# The influence of chromatic background on the photosensitivity of tilapia erythrophores

**DOI:** 10.1242/bio.20146742

**Published:** 2014-01-03

**Authors:** Shyh-Chi Chen, Mark A. W. Hornsby, R. Meldrum Robertson, Craig W. Hawryshyn

**Affiliations:** 1 Department of Biology, Queen's University, Kingston, ON K7L 3N6, Canada; 2 Centre for Neuroscience Studies, Queen's University, Kingston, ON K7L 3N6, Canada

**Keywords:** Chromatophores, Extraretinal photoreceptors, Opsins, Photoresponses

## Abstract

Non-mammalian vertebrates and invertebrates use extraretinal photoreceptors to detect light and perform diverse non-image-forming functions. Compared to well-studied visual systems, the effect of ambient light conditions on photosensory systems of extraretinal photoreceptors is poorly understood. Chromatophores are photosensitive dermal pigment cells that play an important role in the formation of body color patterns to fit the surrounding environment. Here, we used tilapia erythrophores to investigate the relationship between environmental light and chromatophore photoresponses. All erythrophores from three spectral conditions aggregated their pigment granules in UV/short wavelengths and dispersed in middle/long wavelengths. Unlike retinal visual systems, environmental light did not change the usage of the primary opsins responsible for aggregation and dispersion. In addition, short wavelength-rich and red-shifted background conditions led to an inhibitory effect on erythrophore photoresponses. We suggest that, as extraretinal photoreceptors for non-image-forming functions, chromatophores directly adjust their photoresponse sensitivity via changes in opsin expression levels rather than opsin types when environmental light changes.

## Introduction

Surviving in a complex environment requires precise coordination of sensory and signaling systems. As a result, organisms must evolve mechanisms to receive and process input signals and make corresponding responses to biotic and abiotic stimuli. Chromatophores are specialized pigment cells possessing both sensory and signaling characteristics, and they play an important role in animal communication and recognition (Fujii, 2000; Nilsson Sköld et al., 2013). In addition to neural and hormonal regulation, incident light can directly induce color change of chromatophores (Chen et al., 2013; Oshima and Yokozeki, 1999; Sato et al., 2004). Because of this photosensitivity, chromatophore photoresponses might be driven by opsin-based visual pigments (Ban et al., 2005; Chen et al., 2013). Thus chromatophores serve as extraretinal photoreceptors responsible for diverse non-image-forming functions (Shand and Foster, 1999). However, like other extraretinal photoreceptors, how ambient light shapes the photosensory system within chromatophores remains unexplored.

The adaptation of sensory systems facilitates survival in variable environments. Visual systems are able to respond to pressures imposed by environmental changes (Shand et al., 2008). Teleosts, such as deep-sea fish and African cichlids, provide excellent examples of such environmental adaptation. Deep-sea fish possess simple visual systems optimized to detect blue light around 480 nm due to the narrow spectral range of penetrating light in deep sea (Douglas and Partridge, 1997). In cichlid fish dwelling in variable background light conditions, sensory drive is thought to facilitate their color polymorphism and speciation (Seehausen et al., 2008). Nile tilapia juveniles (*Oreochromis niloticus*, an ancestral outgroup to African cichlids) reared in different background light conditions differ in their spectral sensitivity (Hornsby et al., 2013). In the tilapia visual system, seven cone opsins are differentially expressed during development and their maximum absorbance spectra (λ_max_) reported as: SWS1 (360 nm), SWS2b (425 nm), SWS2a (456 nm), RH2b (472 nm), RH2aβ (518 nm), RH2aα (528 nm), and LWS (561 nm) (Spady et al., 2006). In tilapia erythrophores, all seven cone opsins have been detected, which could be correlated to the photoresponse patterns of erythrophores (Ban et al., 2005; Chen et al., 2013). Therefore, erythrophores represent a model particularly suitable for investigating the influence of the light environment on photosensory mechanisms of extraretinal photoreceptors. In this study, we investigated how environmental light conditions modulate the photosensory system of tilapia erythrophores. We employed three chromatic backgrounds to examine the plasticity of erythrophore photosensitivity under different environmental light conditions. The findings lead to a better understanding of the adaptive mechanisms underlying color change of photosensitive chromatophores.

## Results and Discussion

**Fig. 1.**
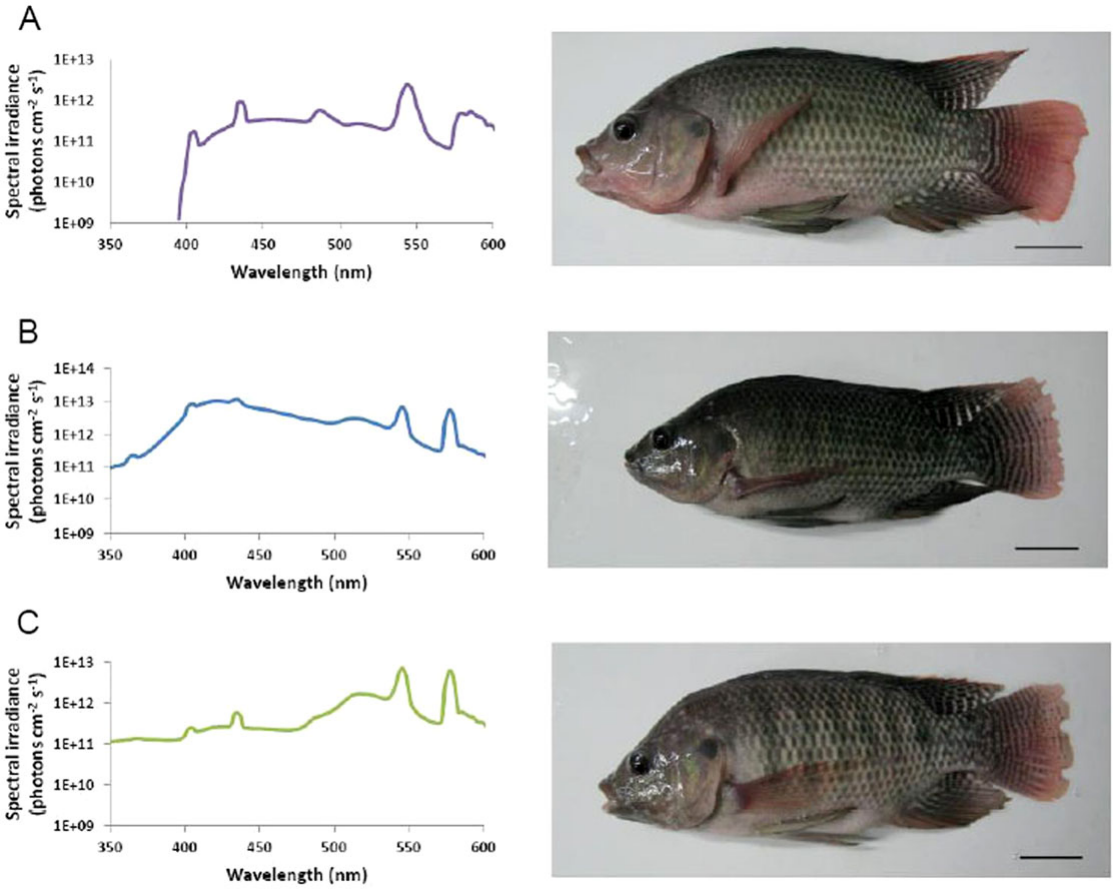
Spectral irradiance for experimental light conditions and the appearance of fish under different light treatments. Tilapia were reared in three spectral backgrounds: (A) broad spectrum, (B) short wavelength-rich, and (C) red-shifted light conditions. After exposure to different light conditions for 2 months, fish showed morphological color change of chromatophores and varied in their appearances. Scale bars: 3 cm.

**Fig. 2.**
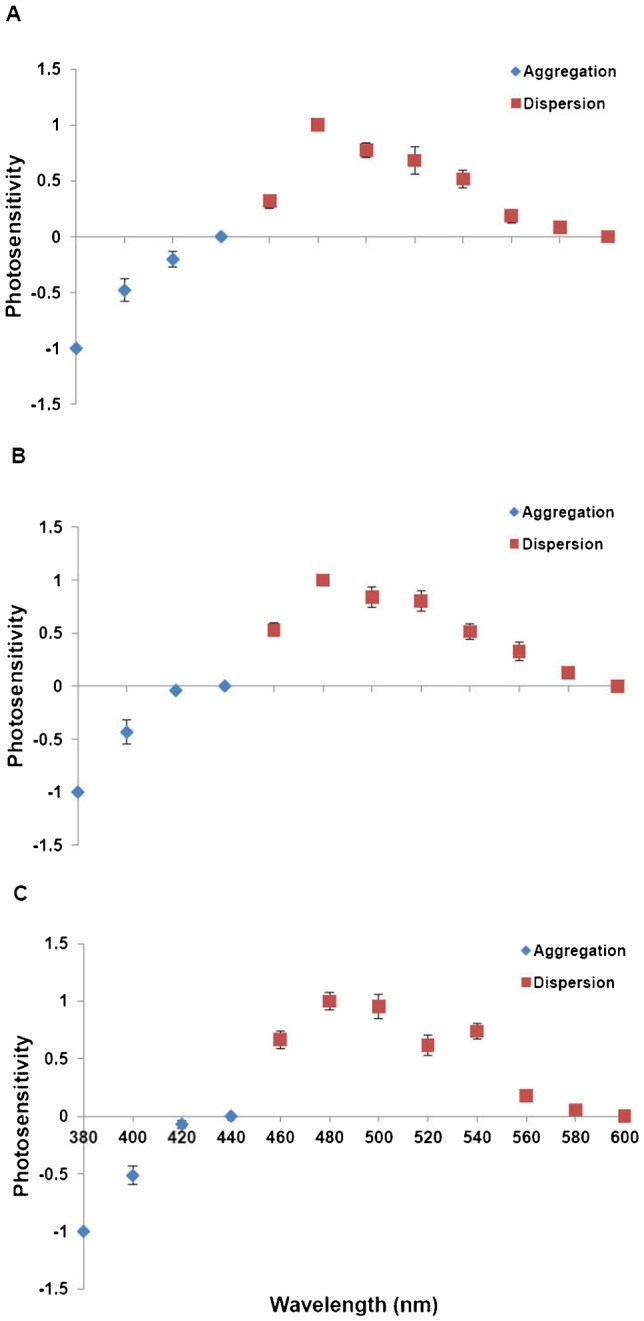
Spectral sensitivities of erythrophores from fish reared in different light conditions. Erythrophores showed biphasic, chromatic photoresponses in all three light conditions: (A) broad spectrum (*n* = 9); (B) short wavelength-rich (*n* = 12); (C) red-shifted (*n* = 11) light conditions. Aggregations occurred in the UV and short wavelengths (380–440 nm; red solid squares), whereas dispersions took place in the middle and long wavelengths (460–600 nm; blue solid diamonds). Two major sensitivity peaks presenting at 380 and 480 nm imply that two opsins were primarily responsible for aggregations and dispersions.

Tilapia erythrophores are photosensitive, aggregating and dispersing in response to light (Chen et al., 2013). Here, we used erythrophores to study how an intrinsic photosensory system is modulated when tilapia undergo spectral changes of the light environment. To determine how the adaptive change of the photosensory system within tilapia erythrophores relates to spectral environments, we employed three different light conditions in the present study. Tilapia reared under broad spectrum light were transferred to a broad spectrum, short wavelength-rich or red-shifted light condition for 2 months (Fig. 1). In response to different backgrounds, fish altered their pigmentation through morphological color change (Fig. 1), which was consistent with a previous study on the same species (Hornsby et al., 2013). Thus, the difference in the appearance of fish should come from the change of chromatophores in size and/or number. We further measured the spectral sensitivity of erythrophores on split-fin tissues isolated from tilapia with three light treatments. Under illumination ranging from 380 to 600 nm, erythrophores translocated inner pigment granules (erythrosomes) in a wavelength-dependent manner. All erythrophores from the three groups showed aggregation in UV and short wavelength (380–440 nm) whereas dispersion took place in middle and long wavelengths (460–600 nm) (Fig. 2). Two major sensitivity peaks present in the spectral sensitivity curves imply that two light-sensitive molecules are primarily responsible for erythrophore photoresponses. Those two peaks appear at 380 and 480 nm, which are close to the λ_max_ of tilapia cone opsins, SWS1 (360 nm) and RH2b (472 nm). Moreover, in the opsin expression profile of tilapia erythrophores, *SWS1* and *RH2* group genes (*RH2b*/*RH2aβ*/*RH2aα*) are detected at a high frequency (Chen et al., 2013). Together, this suggests that SWS1 and RH2b play important roles in erythrophore photoresponses and construct a chromatically-dependent antagonistic mechanism within erythrophores. Therefore, we suggest that with different light treatments, the photosensitive system of erythrophores retains the usage of SWS1and RH2b for their photoresponses. It is noteworthy that in addition to UV and short wavelengths, tilapia erythrophores have been reported to aggregate in long wavelength light (Ban et al., 2005; Sato et al., 2004). The discrepancy between previous studies and ours may be due to different spectral conditions used in the fish culture facility, which could lead to different opsin expression during development.

Besides two primary peaks at 380 and 480 nm, we also found a minor peak at 540 nm in fish from the red-shifted light condition (Fig. 2C). This peak could be due to the rise of an additional opsin within erythrophores. Although we speculate that the opsin present at 540 nm could be the expression of RH2aα (λ_max_ = 528 nm), the mechanism underlying the appearance of this opsin remains unclear. Additionally, novel opsins have been discovered in a variety of extraretinal photoreceptors (Shand and Foster, 1999). Due to their diversity and unclear molecular information in tilapia, we were not able to thoroughly examine the expression of these opsins in erythrophores. Without further investigation on opsin expression, we cannot rule out the possibility that other opsins are expressed and functionally involved in erythrophore photoresponses.

**Fig. 3.**
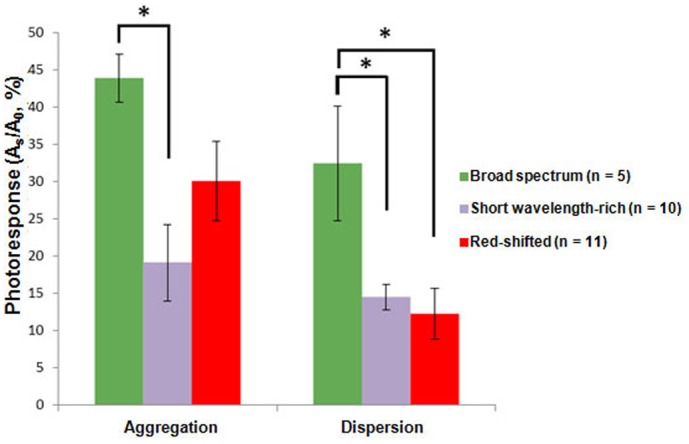
Effect of light conditions on photoresponses of tilapia erythrophores. Photoresponses of erythrophores were measured *in vitro* at the wavelengths where their sensitivity peaks appeared (380 nm for aggregation; 480 nm for dispersion). The erythrophores from short wavelength-rich and red-shifted conditions showed reduced photoresponses compared with cells from broad spectrum condition. A_s_: the change of the pigmented area; A_0_: the maximum capacity of the translocation of erythrosomes. *p<0.05; ANOVA analysis followed by Bonferroni *t*-test. Data are means ± s.e.m.

In killifish, *Fundulus heteroclitus*, melanophores and xanthophores show different motility patterns (i.e. aggregations or dispersions) in response to different background colors (Fries, 1931). This ability to carry out color change may allow the fish to adapt to the alteration of background light conditions, and it can improve with practice (Fries, 1931). Indeed, through cyclical training, zebrafish melanophores enhanced performance of melanosome dispersion by increasing pigmented area and shortening the response time in response to background changes (Hatamoto and Shingyoji, 2008). Thus, this color change process may be processed by a discriminatory center in the brain, suggesting that learning is involved (Fries, 1931; Hatamoto and Shingyoji, 2008). However, since these studies were conducted on whole animals, it remains unclear how photic background conditions influence the photosensitivity of chromatophores per se. To determine if different chromatic treatments will lead to any effect on aggregation and dispersion, we measured the photoresponses at 380 and 480 nm, where the primary peaks appeared. Compared to the group under the broad spectrum light condition, the magnitude of aggregation significantly decreased in the group treated with short wavelength-rich light condition (Fig. 3). On the other hand, both of the groups treated with short wavelength-rich and red-shifted light conditions showed significant reduction in dispersion (Fig. 3). Background adaptation can lead to the alteration of the responsiveness to hormones or neurotransmitters via enzyme activity in the intracellular signaling system of chromatophores (van der Salm et al., 2005). As a result, the change of the erythrophore photoresponses might be due to the effect of backgrounds on internal components of the phototransduction cascade. Alternatively, the change of photoresponses may result from the modulation of the expression level of endogenous opsins. In the visual system, environmental light can regulate photoreception and opsin expression. Recent investigations on the visual systems of black bream and tilapia have shown that fish reared in distinct photic conditions differ in their opsin expression pattern and spectral sensitivity (Hornsby et al., 2013; Shand et al., 2008). The decline in erythrophore photoresponses under different light treatments seems likely to be due to the change of opsin expression levels. Therefore, more light in a particular spectral range could suppress the expression of opsins with λ_max_ within this spectral region although the mechanism giving rise to the inhibitory effect is unknown.

In the present study, tilapia erythrophores produced a biphasic photoresponse pattern in the spectrum ranging from UV to long wavelength light. The interaction between opsins may construct a chromatically-dependent antagonistic mechanism within tilapia erythrophores. Because extraretinal photoreceptors are mainly responsible for the detection of the quality and quantity of light, it seems unnecessary for chromatophores to undertake wavelength tuning by switching opsin types when environmental light changes. As extraretinal photoreceptors, erythrophores function in a similar fashion to color-opponency in the visual system. Aggregations occur at short wavelengths, and dispersions take place at middle and long wavelengths. There is likely a specific benefit for tilapia erythrophores being able to detect changes in the quantity and quality of light. For example, at dawn and dusk the light level is low and only the more sensitive SWS1-driven mechanism is active; therefore, tilapia erythrophores tend to aggregate. To date, extraretinal photoreceptors have been thought to function in non-image-forming tasks; in this sense, tilapia erythrophores are not different. However, with a chromatically antagonistic, or opponent, photosensory system, they could be capable of performing wavelength discrimination to detect subtle changes in environmental light, and of fine-tuning their color output accordingly. Future studies should investigate the change of opsin expression and downstream components in phototransduction under different background conditions.

## Materials and Methods

### Animals

Adult male tilapia *Oreochromis niloticus* were obtained from Northern American Tilapia Inc. (Lindsay, Ontario, Canada). Fish were held in the aquatic facility at a water temperature of 25°C with full spectrum fluorescent lamps (Full Spectrum Solutions, Inc., Jackson, MI, USA) under a 12 h light: 12 h dark photoperiod. All procedures complied with the Canadian Council for Animal Care regulations and the Queen's University Animal Care Committee.

Tilapia were transferred and reared in three isolated 80 L tanks under three spectral backgrounds for two months. To generate different light conditions, full spectrum fluorescent lamps and broad-spectrum blue fluorescent lamps (UV-Blue Actinic lamps; Full Spectrum Solutions, Inc., Jackson, MI, USA) were used for broad spectrum light and short wavelength-rich/red-shifted light conditions, respectively. For each tank, UV-transmissible Plexiglas lids (ACRILYTE, Evonik Industries, NJ, USA) were used and the walls were covered by Black coroplast (Coroplast, Cornwall, ON, Canada). To generate the red-shifted light condition, a yellow-coloured film (Rosco, Markham, ON, Canada) was fixed to the lid to reduce the light at short-wavelength spectral region. The spectral irradiance was measured by a spectroradiometer (QE65000; Ocean Optics, Dunedin, FL, USA) according to the standard protocol as previously described (Chen et al., 2013; Hornsby et al., 2013).

### Measurements of erythrophore photoresponses

Split-fin tissues containing erythrophores were isolated from caudal fins and incubated in PBS (NaCl 125.3 mM, KCl 2.7 mM, CaCl_2_ 1.8 mM, MgCl_2_ 1.8 mM, D-glucose 5.6 mM, Tris-HCl buffer 5.0 mM [pH 7.2] (Ban et al., 2005)) for 15-min dark adaptation before experiments. To examine the photoresponses, tissues were presented with light stimuli generated by a 150 W xenon lamp system and a monochrometer (Photon Technology International, London, ON, Canada). Images were taken by a Qimaging Microimager II CCD camera with QCapture Suite V2.46 software (Qimaging, Burnaby, BC, Canada) and analyzed using Matlab software (Mathworks, Natick, MA, USA) for pixel counts of pigment-covered area of a cell in a series of images. The maximum capacity (A0) of the translocation of erythrosomes was calculated as:
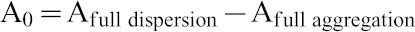
(1)where A_full dispersion_ and A_full aggregation_ denote the pixel counts of each cell at full dispersion and aggregation, respectively.

Erythrophores aggregate in the UV- and short-wavelength spectral regions, while disperse in the middle and long wavelengths (Chen et al., 2013). In order to choose an appropriate stimulating intensity for aggregations and dispersions, response versus intensity (RI) curves were generated. Erythrophores were first presented at 380 nm (12.26 log photons cm^−2^ s^−1^) or 500 nm (13.92 log photons cm^−2^ s^−1^) for 3 minutes, followed by a 3-min darkness to allow cells to completely aggregate or disperse. Subsequently, cells were presented with a 3-min light stimulus at one of the following intensities (for aggregation: 11.67, 11.92, 12.26, 12.62 and 12.86 log photons cm^−2^ s^−1^; for dispersion: 12.87, 13.09, 13.3, 13.52, 13.72 and 13.92 log photons cm^−2^ s^−1^). The intensity used in each cycle gradually increased during the measurements. The change of the pigmented area (A) at an assigned intensity was estimated as:
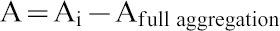
(2)where A_i_ denotes the pixel counts of pigmented area at an assigned intensity (i). The intensities (I_s_) required to reach half-maximal photoresponses (A/A_0_ = 0.5) were used for the following photoresponse assessments (I_s_ for aggregations: 12.37 log photons cm^−2^ s^−1^ and dispersions: 13.31 log photons cm^−2^ s^−1^). Erythrophore photoresponses were measured by means of the procedure mentioned above. To achieve full aggregations or dispersions, cells were presented under illumination at 380 nm (12.26 log photons cm^−2^ s^−1^) or 500 nm (13.92 log photons cm^−2^ s^−1^) for 3 minutes, followed by 3-min darkness. Then, cells were challenged with light stimulus ranging from 380 to 600 nm at I_s_ for 3 minutes. The change of the pigmented area (A_s_) at each wavelength was estimated as:
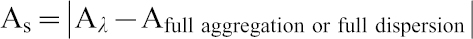
(3)where A_λ_ denotes the pigmented area at an assigned wavelength (λ). The spectral sensitivity was defined as the magnitude of photoresponse (A_s_/A_0_) at a given test wavelength. To minimize the variation between cells, the photosensitivity data were normalized to unity. The photosensitivity curve of erythrophores was generated by mean normalized sensitivity against test wavelengths.
